# Counterfactual Evaluation of Outcomes in Social Risk Decision-Making Situations: The Cognitive Developmental Paradox Revisited

**DOI:** 10.5709/acp-0183-2

**Published:** 2016-03-31

**Authors:** Iván Padrón, María Jose Rodrigo, Manuel de Vega

**Affiliations:** 1 Developmental Psychology, University of La Laguna, La Laguna, Spain; 2 Cognitive Psychology, University of La Laguna, La Laguna, Spain

**Keywords:** risk decision making, counterfactual evaluation of outcomes, social feedback, adolescence

## Abstract

We report a study that examined the existence of a cognitive developmental paradox in the counterfactual evaluation of decision-making outcomes. According to this paradox adolescents and young adults could be able to apply counterfactual reasoning and, yet, their counterfactual evaluation of outcomes could be biased in a salient socio-emotional context. To this aim, we analyzed the impact of health and social feedback on the counterfactual evaluation of outcomes in a laboratory decision-making task involving short narratives with the presence of peers. Forty risky (e.g., taking or refusing a drug), forty neutral decisions (e.g., eating a hamburger or a hotdog), and emotions felt following positive or negative outcomes were examined in 256 early, mid- and late adolescents, and young adults, evenly distributed. Results showed that emotional ratings to negative outcomes (regret and disappointment) but not to positive outcomes (relief and elation) were attenuated when feedback was provided. Evidence of development of cognitive decision-making capacities did also exist, as the capacity to perform faster emotional ratings and to differentially allocate more resources to the elaboration of emotional ratings when no feedback information was available increased with age. Overall, we interpret these findings as challenging the traditional cognitive developmental assumption that development necessarily proceeds from lesser to greater capacities, reflecting the impact of socio-emotional processes that could bias the counterfactual evaluation of social decision-making outcomes.

## Introduction

There is now a broad acknowledgement that counterfactual evaluation of outcomes plays a role in everyday decision making. Upon making the decision and observing the outcomes, people are able to process not only what actually occurred but also an alternative course of events that might have occurred if a different option had been chosen. This complex evaluation requires the cognitive capacity to engage in counterfactual thinking, which is usually accompanied by emotions (Byrne, 2005; Epstude & Roese, 2008; Roese, 2005). Some studies have analyzed counterfactual emotions, such as regret or disappointment, by manipulating the feedback participants saw after making a decision to play certain gambles: full-feedback (regret: participant sees the outcomes from both the chosen and unchosen gamble) versus partial-feedback (disappointment: participant only sees the outcome from the chosen gamble) (Camille et al., 2004).

Other studies have also characterized regret and disappointment by differential agency attribution: personal/controlled agency for regret and relief, external/uncontrolled agency for disappointment and elation (Girotto, Legrenzi, & Rizzo, 1991; Wilkinson, Ball, & Alford, 2015; Wilkinson, Ball, & Cooper, 2010). According to this approach, the emotions of regret and relief typically arise in risk situations (e.g., taking or refusing a drug), where one is, or feels, responsible for the occurrence of a negative or positive outcome that is under one’s control (Connolly & Zeelenberg, 2002; Coricelli & Rustichini, 2010; Ferrell, Guttentag, & Gredlein, 2009; van Dijk & Zeelenberg, 2002; Zeelenberg & Pieters, 2007). By contrast, the emotions of disappointment and elation typically arise in neutral situations (e.g., eating a hamburger or a hotdog), where one is relatively free of self-blame, because the negative or the positive outcome of the decision is appraised as beyond one’s control, such as an accident (Zeelenberg & Pieters, 2007; Zeelenberg, van Dijk, Manstead, & van der Pligt, 2000).

The present study takes this second approach to the study of counterfactual emotions in risk decision-making situations. We examined the adolescents’ and young adults’ decisions involving controlled and uncontrolled events and their counterfactual evaluation of their negative and positive outcomes to induce the respective emotions of regret, relief, disappointment, and elation. Adolescence is a period of increasing risk-taking behavior, including practicing dangerous sports, drinking alcohol, engaging in unsafe experimentation with addictive substances, among others (Vermont Department of Health, 2013). However, the topic of the counterfactual evaluation of outcomes has been largely neglected in the decision-making literature. And yet, counterfactual feelings, such as regret, may help adolescents to prevent risky consequences by making adaptive changes in their behavior for future occasions (Epstude & Roese, 2011; Smallman & Roese, 2009; Wong, Haselhuhn, & Kray, 2012). Moreover, little is known about adolescents’ and young adults’ sensitivity to counterfactually mediated emotions in social situations involving the presence of peers. In these cases, heightened sensitivity to the peer presence has been linked to the adolescent increases in risky decisions, in spite of their acknowledgment of the potential consequences on health (Blakemore & Robbins, 2012). Therefore, it could be the case that the counterfactual evaluation of outcomes is biased in social situations with peer presence, leading to a poor weighing of the consequences. To this aim, the study presents short narratives involving situations in which the presence of peers was made salient in all cases. To better challenge the process of evaluation of outcomes, we also manipulated the presence or absence of health and peer-relevant feedback to examine its impact on the counterfactual evaluation of negative and positive outcomes.

### The Cognitive Developmental Paradox Revisited

The findings of this study may help to demonstrate the possible existence of a cognitive developmental paradox not only in the decision-making process but also in the realm of the counterfactual evaluation of outcomes. Traditional developmental theory presupposes that with age cognitive development proceeds from lesser to greater sophistication, and that increased cognitive skill should decrease the likelihood of participation in risks (Arnett, 1995; Elkind, 1985; Halpern-Felsher & Cauffman, 2001; Vartanian, 2000). The cognitive paradox is that adolescents take more risks than children or preadolescents, even when they have more cognitive decision-making skills (Boyer, 2006). It is similarly perplexing that adolescents do take more risks than adults, but have relatively similar cognitive decision-making capacities, at least in terms of their capacity to analyze risk-taking situations and to estimate the probability of the outcomes (Boyer, 2006). Crucially, adolescents are even able to perceive the negative outcomes associated with a risky decision similarly to adults (e.g., Burnett, Bault, Coricelli, & Blakemore, 2010; Reyna & Farley, 2006).

What happens with regard to the counterfactual evaluation of outcomes in risk decisions? Is there a cognitive developmental paradox, too? The studies on the developmental progression of counterfactual reasoning from childhood to adulthood have shown that although five- to seven-year-old children are able to experience regret and relief (Guttentag & Ferrell, 2008; Weisberg & Beck, 2010, 2012), the ability to experience these emotions continues to develop throughout late childhood and adolescence, suggesting that children’s ability to reason counterfactually is not fully developed in all children before 12 years of age (Habib et al., 2012; Rafetseder & Perner, 2012; Rafetseder, Schwitalla, & Perner, 2013). Therefore, a pure cognitive account would expect that the increase in cognitive sophistication in counterfactual reasoning that is shown to come with the transition from childhood to adolescence, especially from 12 years of age on (e.g., Habib et al., 2012), should lead to a better evaluation of the outcomes. Counterfactual emotions, such as regret, are highly adaptive and can have a significant impact on the reduction of risky decisions in the future (Conner, Sandberg, McMillan, & Higgins, 2006; Richard, van der Pligt, & De Vries, 1996).

There are reasons to suspect, however, that a cognitive developmental paradox could also exist in the counterfactual evaluation of outcomes. According to the dual-processes account (Boyer, 2006; Crone & Dahl, 2012; Somerville, Jones, & Casey, 2010; Steinberg, 2010), adolescents’ decisions appear to be highly sensitive to the presence of socio-emotional cues (e.g., peers) as demonstrated by their increased risk-taking behavior as compared to youth and adults in presence of peers (Gardner & Steinberg, 2005). Similarly, the counterfactual evaluation of outcomes after making a choice could be biased in a salient socio-emotional context (Amsel, Bowden, Cottrell, & Sullivan, 2005). Thus, though there may be some cognitive development in the counterfactual reasoning in adolescent years, the outcome evaluation could be biased since adolescents are confronted not only to negative health consequences but also to potential benefits that are emotionally or socially valuable, such as increasing popularity among peers (Boyer, 2006; Crone & Dahl, 2012; Somerville et al., 2010). To test this possibility, in this study we manipulated the presence or absence of feedback on health and peer popularity.

The existence of a cognitive developmental paradox and its possible explanation according to the dual-process proposal has not been tested in the realm of counterfactual evaluations of decision-making outcomes in social situations. The exception is one recent study examining the ability to experience regret and relief in children, adolescents, and young adults who performed a gambling task in a socio-emotional context of competition, in which they were informed that their outcome would be compared with that of a competitor (Habib et al., 2015). Results showed that in the maximal regret condition (a low loss combined with a high win for the competitor) adolescents did not experience regret, whereas children and young adults did. Under outcome conditions designed to induce relief (in which the competitor obtained a lower outcome than the participant), adolescents and young adults experienced relief, whereas children did not. However, these findings are not conclusive since the gambling task did not depict risk decision-making situations under uncertainty. Only controllable events were included, and all the situations were competitive without a contrasting condition.

### The Present Study

The present study uses analogues to real-life social decision-making by means of the social context decision-making task (SCDT, Rodrigo, Padrón, de Vega, & Ferstl, 2014). This task involves verbal narratives describing situations in which the participants were asked to imagine themselves, accompanied by a peer, either involved in risky/safe choices (e.g., drinking a lot or staying sober) in risk situations, or neutral choices (e.g., eating a hamburger or a hotdog) in neutral situations, and they were told the positive and negative outcomes. Receiving consequences in risk situations involves controllable events since participants may have clear expectations about the possible outcomes of each choice. This is not the case when receiving consequences in the neutral situations where expectations about the outcomes are not clear since they involve uncontrollable events (e.g., accidents). In this way, by manipulating the type of decisions to be made either in risky or in neutral situations as well as the negative or positive outcomes received, we can create the counterfactual conditions to experience the four emotions (regret, relief, disappointment, and elation) in the same study. The study also manipulates the conditions that make the task more or less socio-emotionally salient to examine their impact on the counterfactual evaluation of outcomes. Thus, we manipulated the presence or not of feedback, given after participants were told the outcome of their decision in the task. The feedback includes information on the impact of the consequences on health status and peer popularity on each trial, but also information about the accumulative gains and losses every 10 trials.

**Figure 1.**
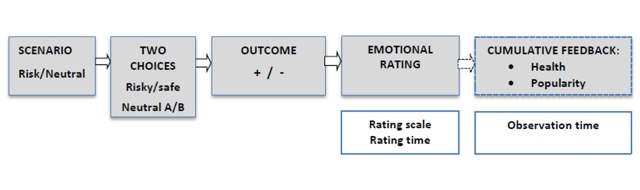
Trial sequences (grey boxes) and measurements recorded (white boxes). Notice that the cumulative feedback (dashed box) is only available in the feedback condition.

Both the risky and the neutral trials involved the same sequence of events presented on the screen as illustrated in Figure 1:

1) A second-person scenario describing “you” as accompanied by a close friend;

2) the two alternative options for the decision-making task in that scenario;

3) the outcome of the choice selected, either positive or negative;

4) the emotional rating scale, where participants had to indicate “how do you feel about what just happened?” using a linear scale from -5 (*extremely bad*) to +5 (*extremely good*), placed at the bottom of the screen; and

5) the feedback information (half of the trials), to inform participants about the gains and losses in health and peer popularity depending on the choice made and the negative or positive consequence received.

The outcomes presented were pre-set by the experimenter following a table of contingencies (Table 1). First, there were gains and losses in health and peer popularity in risk situations, whereas in neutral situations there were gains and losses in health but no gains or losses in peer popularity. The reason is that in real-life situations there are no clear expectations concerning the impact on peer popularity of eating a hamburger or a hotdog. Second, after making a safe choice, participants received a positive outcome in health but not in popularity, since avoiding risks does not help to increase popularity among peers. However, the experimental trials followed by a safe decision were not entered into the analyses since the comparison was made among regret, relief, disappointment, and elation, which are the conditions that are factorially crossed. Another reason is that if we had included in “safe trials” a negative health outcome, then this outcome would have necessarily resulted from uncontrolled events, producing a sort of confounding with the disappointment condition. Finally, another feature of the task is that participants made actual decisions. Thus, in the risk situations participants can be conservative (i.e., choose the safe option over the risky option), which may change the percentage of negative and positive outcomes actually received. By contrast, in the neutral situations the amount of negative outcomes that the participants received after their decisions corresponds to the nominal probability set up by the experimenter because the two choices (A or B) are equivalent in terms of possible outcomes.

**Table 1.** Table of Pre-Set Contingencies, Emotions and Feedback on Gains and Losses in Health and Peer Popularity

Election	Outcome	Emotion	Health	Popularity
Risk	75% Negative	Regret	-30	+10
Risk	25% Positive	Relief	+10	+30
Safe*	100% Positive	Happiness	+30	-30
Neutral	35% Negative	Disappointment	-30	0
Neutral	65% Positive	Elation	+10	0

*Note*. * Not used in the analyses.

Our main goal was to investigate whether providing feedback on health status and peer popularity modulates participants’ performance on the counterfactual evaluation of the outcomes (emotional ratings, rating times, and feedback observation time). We expected lower emotional ratings when this feedback is provided, compared to the no feedback condition. When presenting feedback, the negative peers’ reactions (decrease in popularity) were highlighted, which made more salient the socio-emotional context leading to bias in the evaluation of outcomes. Specifically, the feeling of regret resulting from risky choices and negative outcomes would be lessened in the feedback condition. The reason is that in our task risky choices with negative outcomes, though involving health dangers, were associated to gains in peer popularity. This would not be the case for the feeling of relief resulting from risky choices with positive outcomes both in health and popularity. To support our expectation, the feeling of regret was also attenuated in a gambling task by providing a socio-emotional context of competition with peers (Habib et al., 2015). We also predicted that the emotional effect would be more visible in mid-late adolescents, who are reported to be more sensitive to peer effects (Albert, Chein, & Steinberg, 2013). In turn, we expected that the feeling of disappointment would be stronger than that of elation, but it would not be so affected by feedback conditions, age, or sex, since it is related to serious negative outcomes but derived from uncontrollable events and with no consequences on peer popularity.

Previous studies on gender effects reported that adolescent women are more prone than men to perceive situations as risky (Bohlin & Erlandsson, 2007). In fact, boys and men are less risk averse than women (Borghans, Golsteyn, Heckman, & Meijers, 2009; Van Leijenhorst, Westenberg, & Crone, 2008). In absence of previous evidence, we would expect that women, who are usually more risk averse and presumably more prone to feeling regret, would be more affected than men by the feedback condition by lessening the emotional impact of consequences in risk situations, especially in regret conditions.

Altogether, the results of the present study could help to demonstrate the existence of a cognitive developmental paradox in the counterfactual evaluation of decision-making outcomes to the extent that the presence of feedback with health and socially relevant information could affect this evaluation process, as well as the possible impact of age, and gender effects.

## Method

### Participants

A total of 256 volunteers participated in the study, belonging to four age groups: 64 early adolescents (EA), aged 13-14, 32 female and 32 male, *M*_Age_ = 13.5 years, *SD* = 0.5; 64 mid-adolescents (MA), aged 15-16, 32 female and 32 male, *M*_Age_ = 15.6 years, *SD* = 0.5; 64 late adolescents (LA), aged 17-18 years, 32 female and 32 male, *M*_Age_ = 17.50 years, *SD* = .50, from one public high school and 64 young adults (YA), aged 19-20, 32 female and 32 male, *M*_Age_ = 19.5 years, *SD* = 0.5, from a public university and one public technical school. After explaining the aim of the study to the teaching staff of each academic center and receiving the permission from the officials, students volunteered to participate. Written parental consent was obtained for children and adolescents prior to the assessment session. Written consent was also obtained for adult participants, who also volunteered after receiving information about the research. The procedure was approved by the Committee for Research Ethics and Animal Welfare at the University of La Laguna.

### Task and Procedure

The study used a social context decision-making task (SCDT) involving two types of verbal materials: forty risk situations and 40 neutral situations (Rodrigo et al., 2014). Pilot studies were performed for the elaboration of the verbal materials to select the situations, their choices, and outcomes, using different participants. Sixty-three risk scenarios were written, based on situations selected from the Youth Risk Behavior Survey (Vermont Department of Health, 2013). They belonged to four domains: Behaviors that contribute to unintentional injuries (e.g., jumping into the sea from a high rock), risky sporting practices (e.g., climbing without appropriate equipment), unhealthy behaviors (e.g., competing to demonstrate who can eat more burgers), and alcohol and other drug use (e.g., consuming cocaine). Sixty participants (half adolescents and half young adults of both genders) were asked to report whether they had been involved in or personally witnessed a similar situation or not. Then, they were given examples of risky and safe options for each situation, and asked to rate on a scale of 1 to 5 how dangerous these actions would be for the protagonist. For the neutral situations, 60 neutral options were also created and participants had to choose between the two neutral options. We selected only those situations where each option had a 50% of probability of being selected, with no significant age and gender differences (40 situations). To select the positive and negative outcomes, 120 participants (half of them adolescents and half young adults of both genders) were presented with a list of 128 negative events (e.g., risk situations: “while smoking marijuana you feel dizzy and have to go to the doctor”; neutral situations: “while preparing a snack you cut your finger and bleed profusely”), and 128 positive events (e.g., risk situations: “you enjoy swimming at the beach”; neutral situations: “you enjoy the meal at the restaurant”). The participants rated them on a bipolar scale from -5 (*very negative*) to +5 (*very positive*). The length of the sentences in the scenarios was matched in the number of words and unfamiliar words were avoided in all the scenarios.

Example of a risk situation:

“You are in a disco with your close friend. In the toilet you and your friend meet a guy who offers you cocaine”.

Decision: 1) “You buy it” (risky choice), 2) “You tell him that you are not interested” (safe choice).

Outcomes (risky choice): 1) Negative: “You got very sick and had to go to the hospital”, or 2) Positive: “You had a big ’high’ and felt great”

Outcomes (safe choice): Positive: “You enjoyed dancing with your friends”.

Example of a neutral situation:

“You are in a restaurant with your friend checking the menu for lunch”.

Decision: 1) “You decided to get a hamburger”, 2) “You decided to get a hotdog”.

Outcomes: 1) Negative: “The mayonnaise was spoiled and you got sick and had to go to the hospital”; 2) Positive: “You enjoyed the meal as it was very good”.

Participants received the scenarios of the risk and neutral situations auditorily and the choices and outcomes in written format. The presentation of each piece of information was self-paced, allowing for the recording of chronometric data in addition to the behavioral data. The 80 trials (40 risk and 40 neutral situations) were separated by an inter-trial interval of 5 s, and preceded by a 5-trial practice phase. The stimulus presentation was controlled by means of Cogent 2000, a MATLAB Toolbox for presenting stimuli and recording responses with precise timing.

The task was administered individually to the participants in a quiet room at their Secondary School. They were asked to imagine themselves (“imaging you”) as vividly as possible in each situation accompanied with a close friend and choose between the two alternative actions. They were informed that their decisions would have positive or negative outcomes with more or less impact on their health status and their popularity among friends. Half of the participants, randomly selected from the total sample, were submitted to the feedback condition being informed at the end of every trial (by means of bars diagrams) of the specific gains and losses obtained in peer popularity and health (see Table 1). Every 10 trials they were also informed of the cumulative gains and losses in peer popularity and health, having started the task with 300 points in popularity and 300 points in health status. Finally, all participants were informed that as a bonus for their participation one of them would win a laptop computer in a random draw to be made at the end of the data collection. The duration of the task varied between 20 and 25 min, depending on participants’ response times.

With respect to the procedure, once participants entered a quiet room at the school half of them completed the battery of self-report assessment measures first (the self-report questionnaires were not included in analysis as they contained information that is irrelevant to this study) and then, individually, the SCDT in another room; the other half followed a reversed order (first SCDT and then questionnaires).

### Design and Plan of Analyses

A mixed factorial design was used with age (four groups) or gender (two groups) and feedback (present/absent) as between-participant factors, and type of choices (risky/neutral) and outcome valence (negative and positive) as within-participant factors. The dependent variables were the emotional rating, rating time, and observation time of feedback (just in the feedback condition). Rating time is an index of the cognitive cost allocated to the performing of the emotional rating. Observation time is the time spent watching the feedback information, being an indication of the cognitive effort required to process this information. Four emotional conditions were analyzed resulting from the combination of type of choices and valence of outcomes: (a) risky choice and negative outcomes (regret); (b) risky choice and positive outcomes (relief); (c) neutral choice and negative outcomes (disappointment); (d) neutral choice and positive outcomes (elation). The emotional condition resulting from choosing the safe option was not included in the analyses as it always involved positive consequences and could not be crossed factorially with the other emotional conditions. In all the cases analyses of variance (ANOVAs) were used and effect sizes (eta partial square, η_p_^2^ ) were calculated (*small*: > .01; *medium*: > .06 and *large*: > .14). *T*-test and post hoc Bonferroni corrected comparisons were used when necessary. Data points lying > 3 *SD* from the grand mean of the dependent variables involving time measures in each analysis were considered outliers and were excluded from that analysis (2% of the data as average).

## Results

### Emotional Ratings Following Outcomes

Means and standard deviations of emotional ratings after reading the consequences are shown in Table 2. As expected, means are negative for negative emotions and positive for positive emotions and all significantly differed from zero (*p* = .001), which would suggest that participants experienced regret and disappointment for negative emotion scores and relief or elation for positive emotion scores.

**Table 2.** Means and Standard Deviations of Outcome Measures Under Feedback and No Feedback Conditions

		Risk situations		Neutral situations	
		Regret M (SD)	Relief M (SD)	Disappoint-mentM (SD)	ElationM (SD)
Feedback	Emotion ratings (-5,+5)	-1,38 (1,56)	(1,54)	-1,83 (1,38)	3,06 (1,20)
	Rating times (ms)	2616 (711)	2613 (754)	2782 (650)	2567 (548)
	Observation times (ms.)	2264 (920)	1960 (699)	1641 (516)	1699 (546)
No Feedback	Emotion ratings (-5,+5)	-3,13 (1,4)	1,46 (1,98)	-3,31 (1,13)	3,52 (1,04)
	Rating times (ms)	2991 (778)	2804 (895)	2951 (638)	2718 (508)

**Figure 2.**
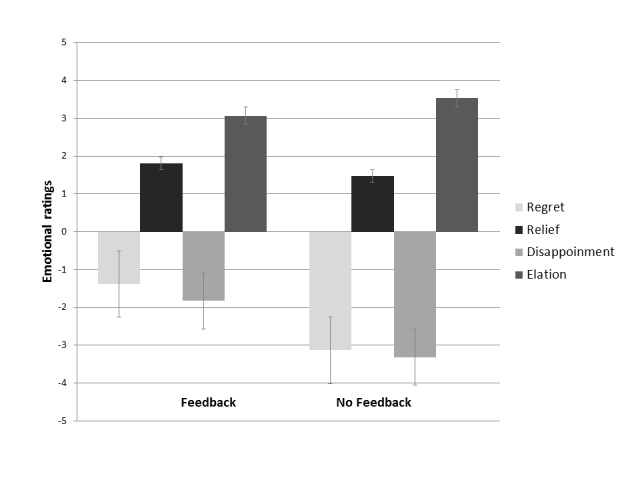
Interaction effects of feedback by outcome valence on the emotional ratings.

Overall, emotional ratings were lower in the feedback condition than in the no feedback condition, *F*(1, 206) = 40.4, *p* = .001, η_p_^2^ = .164 (on average 2.0 and 2.9 respectively). There was a main effect of type of choice, *F*(1, 204) = 69.85, *p* = .001, η_p_^2^ = .255, showing that the emotional ratings were higher in neutral choices than in risky choices. There was also a main effect of outcome valence on emotional ratings, *F*(1, 204) = 1191, *p* = .001, η_p_^2^ = .854), showing higher emotional ratings for negative outcomes (regret and disappointment) than for positive outcomes (relief and elation). However, there was a significant interaction of feedback by outcome valence, *F*(1, 200) = 45.88, *p* = .001, η_p_^2^ =.187 (Figure 2). Thus, emotional ratings for positive outcomes (relief and elation) did not differ between feedback (*M* = 2.43, *SD* = 1.3) and no feedback conditions (*M* = 2.49, *SD* = 1.5). By contrast, emotional ratings for negative outcomes (regret and disappointment) were significantly lower under feedback conditions (*M* =1.60, *SD* = 1.4) than under no feedback conditions (*M* = 3.22, *SD* = 1.2). Significant age effects were not observed.

There was a significant gender effect according to the outcome valence on emotional ratings, *F*(1, 206) = 5.26, *p* = .023, η_p_^2^ = .025. Women reported higher emotional ratings than men when they were told the negative outcome of their decision (regret and disappointment), whereas gender differences were not significant for positive outcomes (relief and elation).

### Emotional Rating Times

Means and standard deviations of emotional rating times after reading the consequences are shown in Table 2. Participants spent more time on the emotional ratings in the no feedback version (2,860 ms) than in the feedback version (2,646 ms), *F*(1, 200) = 7.49, *p* = .050, η_p_^2^ = .020. There was a main effect of outcome valence, *F*(1, 200) = 19.1, *p* = .001, η_p_^2^ = .087, indicating that emotional ratings for negative outcomes took more time than those for positive outcomes. No interaction effects with feedback were observed.

**Figure 3.**
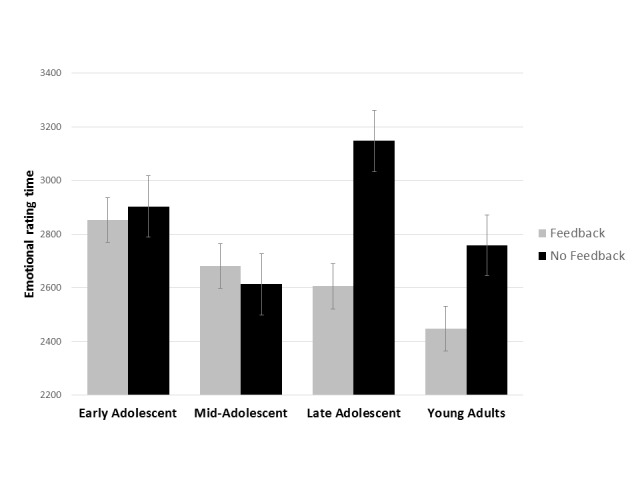
Age differences in emotional rating times by feedback condition.

Age and gender differences were observed in rating times according to feedback conditions. Overall, rating times significantly decreased with age, *F*(3, 204) = 3.52, *p* = .016, η_p_^2^ = .087 , with only the difference between early adolescents and young adults being reliable, *p* < .05, all other comparisons *p* > .10 (EA: *M* = 2,888; *SD* = 481; MA: *M* = 2,745; *SD* = 606 ; LA: *M* = 2,821; *SD* = 569 ; YA: *M* = 2,591; *SD* = 484). However, sensitivity of rating times to feedback conditions also changed with age, *F*(3, 204) = 3.27, *p* = .032, η_p_^2^ = .097, (Figure 3). Overall, emotional rating times were shorter for feedback conditions than for no feedback conditions only in late adolescents (*p* < .001) and young adults (*p* < .05).

There was an interaction of gender by feedback and type of choice, *F*(1, 204) = 4.37, *p* = .038, η_p_^2^ = .021. Simple effects showed that women’s rating times in risky choices (regret and relief) were significantly shorter in the feedback condition than in the no feedback conditions (*p* < .01). Men’s rating times in neutral choices (disappointment and elation) were significantly shorter in the feedback condition than in the no feedback condition (*p* < .05).

### Observation Times

The last set of analyses was performed in the feedback condition only, since the dependent variable was the observation time of feedback information (see Table 2). Participants spent more time inspecting the feedback information in risky choices (2,112 ms) than in neutral choices (1,670 ms), *F*(1, 115) = 81.67, *p* = .001, η_p_^2^ = .415. and after receiving negative outcomes (1,952 ms) as compared to positive outcomes (1,829 ms), *F*(1, 115) = 4.97, *p* = .029, η_p_^2^ = .028. Both effects were qualified by a type of choice × outcome valance interaction, *F*(1, 111) = 13.24, *p* =.001, η_p_^2^ = .103, showing that the above difference was significant in the risky choices (regret > relief, *p* = .001) but not in the neutral choices (disappointment = elation, *p* > .10).

Overall, the observation times decreased with age, *F*(3,115) = 3.09, *p* = .030, η_p_^2^ = .075, with the difference being reliable between early adolescence and mid-adolescence (*p* = .024), late adolescence (*p* = .007) and young adults (*p* = .024), (2,167 ms, 1,833 ms, 1,771 ms, 1,831 ms, respectively).

## Discussion

This study examined the existence of a cognitive paradox in the counterfactual evaluation of risk decision making by means of exploring the impact of gains and losses in health status and peer popularity, as well as age and gender effects. As expected, the presence of feedback in risk situations determines an attenuation of the participants’ emotional experience derived from outcomes as compared to the no feedback conditions, as shown by the lower emotional ratings, and shorter rating times in the former condition. No effect was obtained from the manipulation of feedback in the neutral situations, as expected. Moreover, what seems to be specifically affected in risk situations are the emotions of regret and disappointment which were attenuated in the feedback condition as compared to the no feedback condition, whereas emotions linked to positive outcomes (relief and elation) were not affected by the presence or absence of feedback. This is remarkable, since overall the processing of negative outcomes demands more effort and provokes higher emotional arousal than the processing of positive outcomes, as suggested by higher emotion ratings, and longer rating times. The impact of feedback on risky choices is not likely to be due to the instructions received at the beginning of the task, since in both the feedback and no feedback versions participants were informed that their decisions would have positive or negative consequences with more or less impact on their health status and their popularity among friends. Also it is not likely to be due to a game-like effect according to which participants tend to disregard the task information waiting for the final feedback, since the emotional attenuation is confined to the negative consequences but not to the positive ones.

As expected, participants evaluating the outcomes in the feedback condition were less sensitive to regret feelings as compared to relief feelings (Habib et al., 2015), both derived from risky choices, suggesting that they decreased their avoidance of harm, probably due to the positive impact of negative consequences in peer popularity. However, the counterfactual evaluation associated to the feeling of regret involves more attention demands than that of the feeling of relief, since observation times of feedback information were larger for regret as compared to relief conditions. Probably, participants had to pay comparatively more attention to the conflicting information presented involving health losses and popularity gains in regret conditions, whereas in relief conditions both aspects involve gains.

Unexpectedly, participants were also less sensitive to disappointment feelings under feedback conditions, as compared to elation, which suggests that the surprise effect provoked by non-controlled negative circumstances (Zeelenberg & Pieters, 2007) is also attenuated in this condition. In other words, participants seem to decrease their aversion to ambiguity (e.g., Weber & Tan, 2012), even when feedback involved only health risks. However, this is a “short life” attenuation effect since by the time participants are facing the feedback information, observation times did not differ for disappointment and elation conditions. Probably, there are not many lessons to be learnt from having experienced outcomes under uncontrolled circumstances, as there is no chance to undo what has happened. In favor of this interpretation, previous studies have shown that disappointment, as compared to regret, is relatively free of self-blame and does not lead to behavior change (Zeelenberg et al., 2000; Zeelenberg & Pieters, 2007).

Emotional ratings to outcome information did not vary across age groups, in line with previous results indicating that the children’s ability to reason counterfactually is already in place after 12 years of age (Rafetseder et al., 2013; Rafetseder & Perner, 2012). In fact, previous decision-making studies have found age differences in adolescents’ counterfactual emotions but confined to the comparison to child or adult groups, which respectively were younger and older than our participants’ age groups (Burnett et al., 2010; Habib et al., 2012). Also their results are hardly comparable to ours as they followed a different procedure to elicit counterfactual and non-counterfactual emotions in gambling situations. In our study, rating times and observation times of feedback information were sensitive to developmental effects, since overall there were shorter times with age, probably due to improvements in executive functioning (Crone, 2009; Schiebener, García-Arias, García-Villamisar, Cabanyes-Truffino, & Brand, 2014). However, sensitivity of rating times to feedback conditions changed with age, since emotional rating times were shorter for feedback than for no feedback conditions only in late adolescents and young adults. This age-related effect qualified a general trend observed, showing that counterfactual evaluations made in absence of feedback were more costly than in presence of feedback, suggesting that in absence of explicit task information participants have to rely upon their own cognitive resources to evaluate the outcomes. It seems that late adolescents and young adults were more able to cope with this extra cognitive demand than early and mid-adolescents.

Gender effects on emotional ratings showed that women experienced more emotional intensity than men when confronted with negative outcomes (regret and disappointment) but not when facing positive outcomes, with no feedback effects. That means that overall women become more emotionally activated than men not only when faced with the prospect of receiving negative results but also when actually experiencing harmful consequences (Bohlin & Erlandsson, 2007; Clark et al., 2008). However, the interaction of gender by feedback and type of choice on emotional rating times showed that the awareness of health losses and peer benefits led women to spend shorter times in the risk decisions (i.e., to become less “risk averse”), whereas they led men to spend shorter times in the neutral decisions (i.e., to become less “ambiguity averse”). However, all gender results were weak so they deserve further exploration.

In keeping with the ecological validity of the task we are aware of two possible limitations of the study. First, feedback information differs under risk (health and peer popularity information) and neutral (only health information) situations. Introduction of an arbitrary weight towards gains and losses in peer popularity depending on the neutral choices, instead of a “zero” score, could have affected results in unknown ways. However, we managed to perform the analyses and to draw the main conclusions from the comparisons performed within each type of choice (risk or neutral). Second, it would be interesting to have included a measure of executive functioning to support our interpretation of decreasing times with age in the counterfactual evaluation of outcomes. Third, arguably using real-life scenarios would have entailed some misunderstandings in the interpretation of risk and neutral situations. To keep this possibility at minimum, we have performed pilot studies to elaborate verbal material that could be comparable across ages and genders. We think that simulation of real-life situations is worthwhile to increase the participants’ chances of visualizing the course of actions and foreseeing their consequences, and to facilitate the participants’ actual engagement in counterfactual-related emotional states.

In conclusion, this study demonstrated that the cognitive developmental paradox also existed when considering the counterfactual evaluation of outcomes in a salient socio-emotional context. Evidence of development of cognitive decision-making capacities does exist, as young adults were more capable of performing faster emotional ratings than early adolescents. Moreover, late adolescents and young adults were able to differentially allocate more cognitive resources to the counterfactual evaluation than younger participants in situations when no feedback information is available. However, the paradox exists since across the age groups enhancing the health and the socially relevant consequences of the choices by means of feedback negatively affected the counterfactual evaluation in risk situations by attenuating the emotional sensitivity to the outcomes of the choices, especially the negative ones. This could be potentially damaging since there would be less chances to anticipate regret or to avoid the previously chosen option in the future, two cognitive capacities that are already present in older children (Guttentag & Ferrell, 2008; O’Connor, McCormack, & Feeney, 2014). Altogether, the present findings challenge the traditional cognitive developmental assumption that development necessarily proceeds from lesser to greater capacities and revealed the importance of socio-emotional processes in the counterfactual evaluation of social decision-making outcomes.
